# Breast cancer type 1 and neurodegeneration: consequences of deficient DNA repair

**DOI:** 10.1093/braincomms/fcab117

**Published:** 2021-05-27

**Authors:** Emily Leung, Lili-Naz Hazrati

**Affiliations:** 1 Department of Laboratory Medicine and Pathobiology, University of Toronto, 1 Kings College Cir, Toronto, ON M5S 1A8, Canada; 2 The Hospital for Sick Children, 555 University Ave, Toronto, ON M5G 1X8, Canada

**Keywords:** DNA damage, DNA repair, senescence, BRCA1, neurodegeneration

## Abstract

Numerous cellular processes, including toxic protein aggregation and oxidative stress, have been studied extensively as potential mechanisms underlying neurodegeneration. However, limited therapeutic efficacy targeting these processes has prompted other mechanisms to be explored. Previous research has emphasized a link between cellular senescence and neurodegeneration, where senescence induced by excess DNA damage and deficient DNA repair results in structural and functional changes that ultimately contribute to brain dysfunction and increased vulnerability for neurodegeneration. Specific DNA repair proteins, such as breast cancer type 1, have been associated with both stress-induced senescence and neurodegenerative diseases, however, specific mechanisms remain unclear. Therefore, this review explores DNA damage-induced senescence in the brain as a driver of neurodegeneration, with particular focus on breast cancer type 1, and its potential contribution to sex-specific differences associated with neurodegenerative disease.

## Teaser

DNA damage and deficient DNA repair are well established features of neurodegenerative diseases. This review highlights how the dysregulation of a DNA repair protein encoded by breast cancer type 1 promotes the onset of cellular senescence and contributes to brain dysfunction as it drives neurodegeneration.

## Graphical Abstract

**Graphical Abstract:**
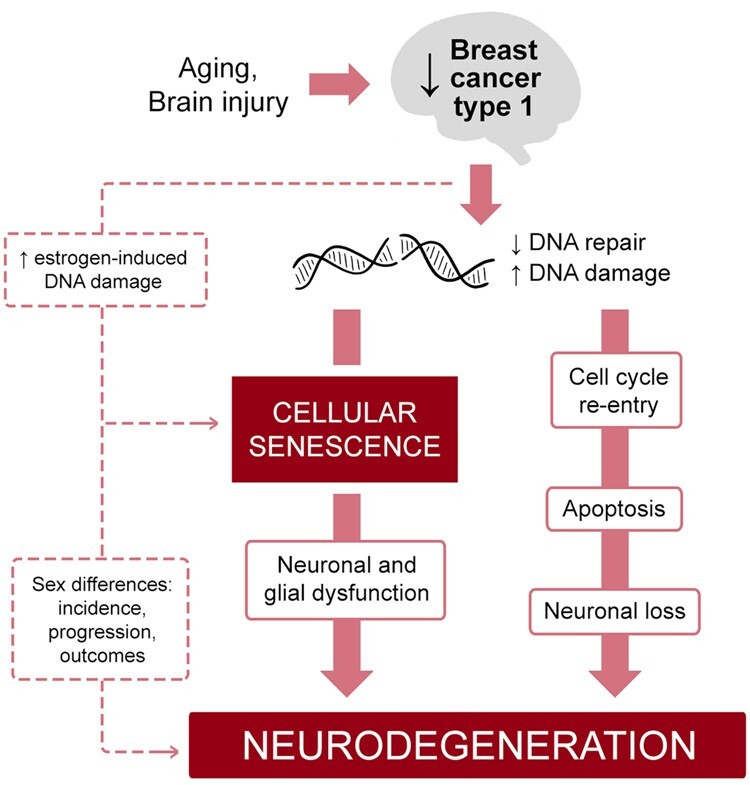

## Introduction

Throughout life, the brain undergoes many changes that can impact cognition and overall brain health. With normal ageing, the brain experiences brain atrophy and white matter degeneration due to a myriad of factors that can negatively affect cognition, such as neuroinflammation, oxidative stress, hypoperfusion and myelin loss.^1–4^ Independent of age, the brain can also become susceptible to neurodegeneration from changes that take place following brain injury. In the context of traumatic brain injury, induction of oxidative stress, increased inflammation, reduced cerebral blood flow, cell death and metabolic dysfunction^5–7^ can occur, leading individuals to undergo structural alterations that can result in reduced cognitive function. As a result, the onset of neurodegeneration is associated with several risk factors and is not exclusive to ageing. Moreover, the occurrence of neurodegenerative diseases can be familial or sporadic. Familial cases only make up a small percentage of cases and depend on the heritability of genetic mutations, predisposing individuals to an earlier onset of the disease.^8^ Sporadic cases, however, make up the majority of cases, with their underlying causes to be much more elusive.

When investigating potential causative mechanisms underlying neurodegeneration, abnormal protein aggregation has been a predominant area of study. Several neurodegenerative diseases are characterized primarily by toxic protein aggregation that contributes to cognitive decline.^9^^,^^10^ These proteins function to propagate disease pathology by promoting aggregation in neighbouring cells in a prion-like manner.^11–14^ However, it is important to note that although the contribution of toxic protein aggregation in neurodegeneration is evident, protein aggregation is not always sufficient to cause neuronal loss.^15^^,^^16^ Alternatively, the brain is particularly susceptible to damage by oxidative stress due to its high metabolism, high lipid content vulnerable to peroxidation and low neuron antioxidant levels.^17^^,^^18^ Numerous studies have implicated oxidative stress in the progression of neurodegenerative diseases,^19^^,^^20^ reporting that protein aggregates can induce oxidative stress, and that conversely, oxidative stress can promote protein aggregation.^21–23^ Indeed, excessive oxidative stress can damage the brain through various mechanisms: DNA/RNA oxidation,^24^ mitochondrial dysfunction,^25^^,^^26^ glial cell activation^27^ and initiation of cell death pathways.^28^ However, antioxidant treatments have shown little efficacy in treating neurodegenerative diseases.^19^ Therefore, abnormal protein aggregation and oxidative stress have been heavily studied in relation to neurodegeneration, but their lack of causation prompts other upstream mechanisms to be explored.

Researchers have documented evidence suggesting that the accumulation of DNA double-stranded breaks and deficient DNA repair may precede pathology present in several neurodegenerative diseases.^29–34^ Consequently, excess DNA damage without sufficient repair has been reported to induce cellular senescence,^35^^,^^36^ resulting in cellular and tissue dysfunction capable of promoting neurodegeneration. Breast cancer type 1 (BRCA1), a key DNA repair protein, has been associated with senescence as well as neurodegenerative diseases, however, its specific mechanisms remain unclear. Thus, this review will explore deficient DNA repair and DNA damage accumulation as a driver of neurodegeneration through DNA damage-induced senescence, with a specific look at BRCA1 as a major contributor to the process.

## Deficient DNA repair and DNA damage accumulation

Defective DNA repair and DNA damage accumulation are well established features of neurodegeneration. As part of the DNA damage response, cells have several repair pathways available to repair different lesions. For single-stranded breaks, lesions caused by base alterations without a helical distortion are repaired via base excision repair, while those with a helical distortion are repaired via nucleotide excision repair.^37^ As previously mentioned, one cause of DNA damage that contributes to neurodegeneration is oxidative stress. Lesions caused by oxidative damage are commonly repaired by base excision repaor.^38^ Indeed, in neurodegenerative diseases, such as Alzheimer’s disease that are highly associated with oxidative stress, deficiencies in base excision repair have been identified.^39^ An impairment of oxidative DNA lesion repair has also been reported in Huntington’s disease.^40^ However, similar deficiencies are not seen throughout different neurodegenerative diseases. Alternatively, fibroblasts isolated from patients with Parkinson’s disease have shown impairments in nucleotide excision repair.^41^ In the context of double-stranded breaks, lesions are repaired by homologous recombination or non-homologous end joining, and can occur during replication and recombination, as a consequence of stressors, such as oxidative stress and genotoxic agents, and from the conversion of single-stranded breaks to double-stranded breaks.^42^^,^^43^ Homologous recombination relies on sequence homology and a repair template available during replication to achieve accurate repair, whereas non-homologous end joining ligates exposed ends back together with less reliance on sequence for a rapid, but potentially more error-prone repair.^44^ In the brain, neural progenitors and stem cells rely more heavily on homologous recombination as actively dividing cells, while neurons resort to non-homologous end joining as post-mitotic cells.^43^ Thus, double-stranded breaks are particularly detrimental to the brain due to limited accurate repair, and leave neurons more vulnerable to the consequences of DNA damage that could lead to degeneration. In support of this, DNA double-stranded breaks and DNA damage response defects have been associated with several neurodegenerative diseases, including Alzheimer’s disease,^45^^,^^46^ Parkinson’s disease,^47^^,^^48^ spinocerebellar ataxia type 1^49^ and 3,^50^ amyotrophic lateral sclerosis^51^^,^^52^ and Huntington's disease.^53^^,^^54^ Moreover, neuronal levels of DNA damage have been shown to correlate with patient Mini-Mental State Examination scores, further affirming a link between the DNA damage response and cognitive impairment.^55^ Interestingly, studies have shown double-stranded break accumulation to not only contribute to the progression of neurodegeneration, but to precede other recognized neuropathological features as well.^30–33^

It has been reported that DNA damage affects neurons in a region-specific manner, suggesting differential neuronal vulnerability throughout the brain with age.^56^^,^^57^ In neurodegenerative diseases, the hippocampus, neocortex and striatum, are some of the brain regions most affected in causing functional decline. With ageing and neurodegeneration, neurons in these regions not only show increased DNA damage in mouse brains,^45^^,^^58^ but also appear to be preserved as opposed to undergoing cell death.^37^ This preservation phenotype, despite an accumulation of DNA damage, suggests that an alternative mechanism may be driving cognitive decline associated with ageing and neurodegeneration prior to inducing neuronal death.

## DNA damage-induced senescence: a driver of neurodegeneration

### Cellular senescence and brain dysfunction

Under certain circumstances, a cell can be driven to undergo cellular senescence or apoptosis.^59^ Cellular senescence is a state of permanent cell cycle arrest useful for preventing the proliferation of damaged cells, and is a recognized feature of ageing and age-associated pathologies.^60^ Characterized by changes in protein homeostasis, morphological and epigenetic modifications, alterations in cellular metabolism, and the adoption of a senescence-associated secretory phenotype that induces an inflammatory phenotype,^61^^,^^62^ this sustained state will ultimately impact cell and tissue function.^59^^,^^61^ Indeed, an accumulation and persistence of DNA damage have been reported as a feature of senescence in both human cells and ageing mice.^35^ Stressors that induce DNA damage or oxidative stress seem to operate in a dose-dependent manner, where lower dosages are associated with senescence and higher doses are associated with apoptosis.^63^^,^^64^ However, some DNA damage-inducing agents have been associated with senescence regardless of dose, suggesting that the type of DNA damage may also influence how a cell responds to stress.^59^^,^^63^ In addition to select cells undergoing senescence due to stress, senescence can be prompted by and maintained in neighbouring senescent cells via paracrine signalling.^65^

Owing to senescence being a state of permanent cell cycle arrest, it is more often associated with mitotic cells. As a result, senescence in post-mitotic cells, such as neurons, has been studied to a much lesser extent. However, studies have speculated that neurons indeed undergo senescence and exhibit several markers and phenotypes characteristic of senescence.^36^^,^^66^ More specifically, aged neurons *in vitro* have been shown to adopt a senescent-like phenotype in response to DNA damage, and are characterized by increased levels of pro-inflammatory cytokines, senescence-associated beta-galactosidase, oxidative damage, and double-stranded break accumulation.^36^ Cultured hippocampal neurons from rats exhibited increasing amounts of senescence-associated beta-galactosidase with age as well as morphological changes associated with senescence.^67^ With senescence-associated changes in gene expression and morphology, the capacity for proper neuronal function is likely altered in these neurons and contributes to cognitive decline.

Other cell types in the brain have also been documented to undergo senescence or adopt a senescent-like phenotype with age an9d neurodegeneration,^60^ including astrocytes,^68^ microglia,^69^ oligodendrocytes,^70^ endothelial cells^71^^,^^72^ and neural stem cells.^73^ A study investigating senescent hallmarks at chronic time points in a neuron and glial cell co-culture found increases in markers of senescence, DNA damage and oxidative stress, accompanied by a loss of neurons and activated glial cells.^74^ As cells in the central nervous system work collectively to promote proper brain function, researchers have suggested that rather than neurons undergoing senescence, the features of senescence seen in neurons may be attributed to surrounding senescent glial cells that subsequently affect neurons.^66^^,^^75^ For instance, astrocytes regulate the uptake of excess glutamate in the synapse to prevent excitotoxicity.^76^ After inducing senescence by x-irradiation, astrocytes exhibited changes in gene expression that included an upregulation in pro-inflammatory genes and a downregulation in those associated with regulating glutamate. As a result, when co-cultured with neurons in the presence of glutamate, impaired glutamate uptake by senescent astrocytes was followed by increased neuronal death.^76^ Although this mechanism may likely contribute to neurodegeneration, further studies characterizing the effects of senescent glial cells on neurons are needed.

### DNA repair and DNA damage following brain injury

In addition to the onset of senescence in the brain, cellular senescence has been implicated in several neurodegenerative diseases, such as Alzheimer’s disease, Parkinson’s disease, Down Syndrome and multiple sclerosis,^60^^,^^61^ further supporting the role of cellular senescence in driving neurodegeneration. Indeed, in a tau-dependent neurodegenerative mouse model, clearance of accumulated senescent astrocytes and microglia was able to preserve cognition by preventing gliosis and neuron degeneration.^77^ Independent of age, senescence can also occur following brain injury. Previously, our lab identified an accumulation of DNA damage as a marker of brain damage in individuals with a history of mild traumatic brain injury.^34^ In addition, we performed a case series on brains of professional athletes with a history of mild traumatic brain injury post-mortem to investigate DNA damage-induced senescence pathways. Analysis showed an upregulation of senescence-associated gene expression and a downregulation of DNA repair genes, with increased DNA damage in brains with mild traumatic brain injury compared to control brains with no mild traumatic brain injury history.^78^ A recognized marker of double-stranded breaks was identified in glial cells, namely ependymal cells, oligodendrocytes and astrocytes, in mild traumatic brain injury brains but not controls. Other observations included senescence-associated morphological changes identified in astrocytes with DNA damage, and changes in neuronal gene expression associated with neurodegeneration.^78^ These results suggested DNA repair deficiency in the brain may confer susceptibility for DNA damage-induced senescence and encourage the onset of neurodegeneration following mild traumatic brain injury.^78^ These findings not only demonstrate cellular senescence driven by DNA damage as a contributing factor to neurodegeneration, but also the capacity of mild traumatic brain injury to model sporadic neurodegeneration.

### Deficient DNA repair and neurodegeneration

With increasing evidence supporting neurodegeneration driven by DNA repair deficiency potentially through DNA damage-induced senescence in the brain, several DNA repair proteins have been studied. SIRT6 (sirtuin 6), a histone deacetylase, is involved in several DNA repair pathways, including double-stranded break repair, base excision repair and nucleotide excision repair.^79^ When deleted from mice specifically in the brain, mice presented with a neurodegenerative phenotype, consisting of impaired learning, DNA damage accumulation and increased apoptosis.^79^^,^^80^ With more emphasis on double-stranded break repair, ataxia-telangiectasia mutated is a kinase that acts as an upstream sensor for DNA damage and gets recruited to sites of double-stranded breaks to initiate double-stranded break repair.^43^ Mutations in ataxia-telangiectasia mutated result in ataxia telangiectasia, a neurological disorder characterized by cerebellar atrophy, motor function impairment and white matter degeneration.^4^^,^^81^ The MRN complex, consisting of MRE11 (meiotic recombination 11), RAD50 (DNA repair protein RAD50) and NBS1 (Nijmegen breakage syndrome 1), is involved in DNA damage sensing and was found to be expressed at substantially lower levels in cortical neurons of Alzheimer’s disease brain samples compared to control samples.^82^ p53 (tumour protein p53), a transcription factor involved in mediating DNA damage repair, has been shown to aggregate and mislocalize in Alzheimer’s disease brain, causing a reduction in DNA damage responders regulated by p53 and an increase in DNA damage.^83^ BRCA1, a protein involved in both double-stranded break repair pathways, when knocked down in mice resulted in learning and memory impairments, in addition to increased levels of DNA damage.^84^ The persistence of DNA damage response signalling as a result of insufficient DNA repair is a promising avenue to explore as an inducer of senescence that can drive neurodegeneration. Indeed, disrupting the expression of several proteins involved in the DNA damage response were able to ameliorate functional deficits associated with neurological symptoms in several neurodegenerative disease models.^85^

Knowing the toxicity of double-stranded breaks and how compromised genomic integrity can induce DNA-damage induced senescence in the brain, BRCA1 is of particular interest. A study by Suberbielle et al.^84^ reported decreased levels of BRCA1 in an Alzheimer’s disease mouse model compared to wildtype, but similar observations were not seen for other DNA repair proteins. Moreover, levels of BRCA1 in hippocampal neurons were reduced in post-mortem brains of Alzheimer’s disease and mild cognitive impairment patients compared to control patients with no cognitive deficits.^84^ Further experiments investigating mice with a BRCA1 knockdown in the brain showed an increase in neuronal double-stranded breaks and impairments in memory and learning, which were exacerbated in Alzheimer’s disease mice.^84^ In another study investigating the effects of a BRCA1 knockout in embryos, researchers found that these embryos exhibited increased expression of p53 and p21 (cyclin-dependent kinase inhibitor 1), followed by elevated levels of senescence-associated beta-galactosidase at later stages of development, suggesting that DNA damage accumulation due to BRCA1 deficiency may cause premature senescence.^86^ BRCA1 was also downregulated in post-mortem brains with a history of mild traumatic brain injury exhibiting an upregulation of several markers of senescence.^78^ Given the implications of BRCA1 in neurodegeneration and stress-induced senescence, the following sections will further explore BRCA1, the DNA repair pathways in which it operates, and its association with neurodegeneration.

## Breast cancer type 1

### Maintaining genomic integrity

**Figure 1:**
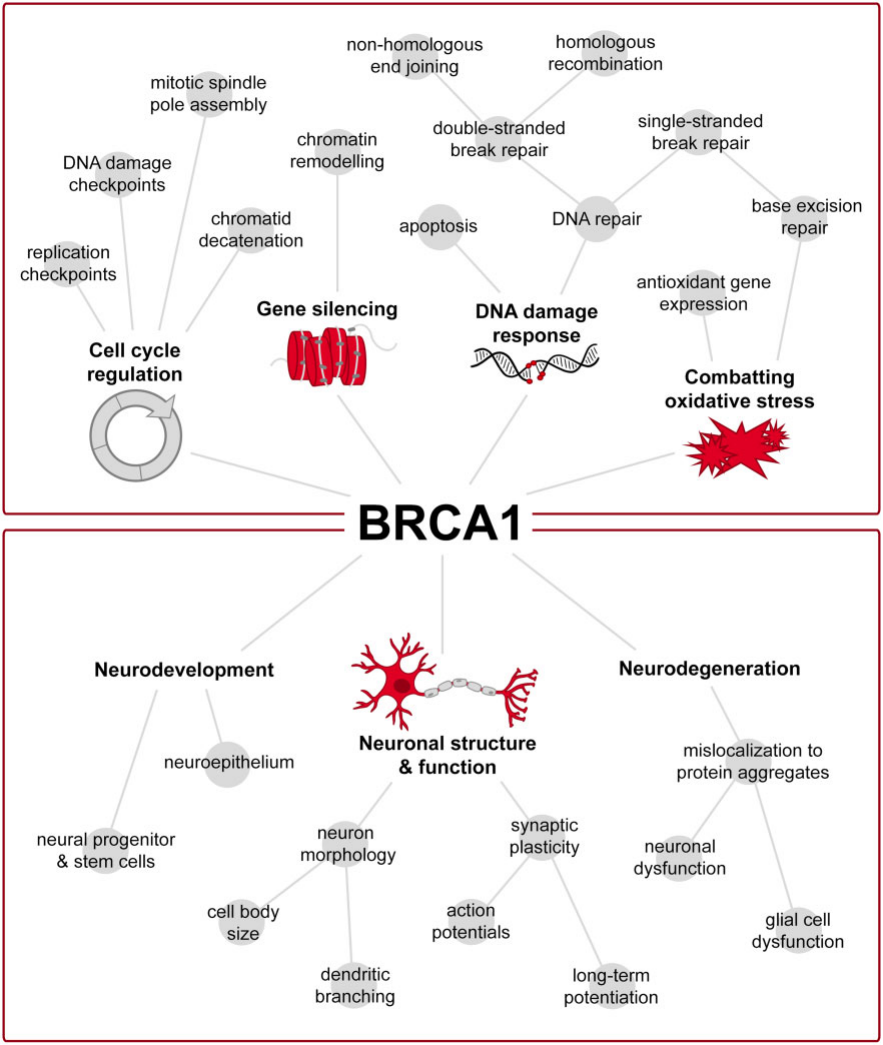
**Functions of BRCA1 in genomic stability and within the brain. BRCA1 maintains genomic integrity through its various roles in cell cycle regulation, gene silencing, protecting against oxidative stress, and the DNA damage response (upper panel).** Within the cell cycle, BRCA1 is involved in both replication and DNA damage checkpoints throughout interphase, and contributes to mitotic spindle pole assembly and sister chromatid decatenation during mitosis. BRCA1 interacts with chromatin and promotes gene silencing through post-transcriptional modifications. To combat oxidative stress, BRCA1 has been shown to promote upregulation of antioxidant gene expression, as well as upregulation of specific proteins in the base excision repair pathway to protect against oxidative lesions. As part of the DNA damage response, BRCA1 serves as a DNA repair protein in multiple repair pathways, and also regulates apoptosis in response to DNA damage. In addition to its role in maintaining genomic stability, BRCA1 also has an integral role in the brain, which has been studied to a lesser extent (lower panel). In particular, BRCA1 plays a proliferative role in the neuroepithelium throughout neurodevelopment with more specificity towards neural progenitor and stem cells later in life. During adulthood, BRCA1 is involved in maintaining neuron morphology and synaptic plasticity, with functions in action potential firing and long-term potentiation involved in learning and memory. Dysregulation of BRCA1 has also been seen in several neurodegenerative diseases, where its mislocalization to pathological lesions likely contributes to neuronal and glial cell dysfunction.

*BRCA1* has been widely recognized for conferring increased susceptibility for breast and ovarian cancer in individuals carrying a mutation in the gene. A prospective cohort study reported the cumulative risk of breast cancer by age 80 to be 72% for *BRCA1* mutation carriers, whereas that of ovarian cancer was reported to be 44%.^87^ Individuals with *BRCA1* mutations not only have a substantial increased risk for breast and ovarian cancer, but an increased risk for other cancers, such as pancreatic and cervical cancer as well. Underlying the reputation of *BRCA1* in breast and ovarian cancer, BRCA1 primarily functions in the nuclear compartment as a tumour suppressor, and participates in various cellular processes, including cell-cycle checkpoints, DNA repair, antioxidant responses and gene silencing (Fig. 1).^88–90^ BRCA1 most commonly exists as a heterodimer to ensure its stability,^91^ but can form several complexes through its N-terminal RING (really interesting new gene) zinc finger domain and BRCT (BRCA1 C Terminus) repeats located at its C-terminal region.^88^

Cell cycle regulation is one facet in which BRCA1 functions to maintain genomic integrity. Functioning through complexes, BRCA1 partakes in mitotic spindle pole assembly and regulation of replication checkpoints.^91–93^ Moreover, BRCA1 also participates in mediating DNA-damage cell cycle checkpoints^93^^,^^94^ and the decatenation of sister chromatids during replication^95^ by regulating post-transcriptional modifications of downstream proteins.

To combat oxidative stress, BRCA1 is involved in regulating the expression of several antioxidant genes. Overexpression of BRCA1 in prostate and breast cancer cell lines resulted in upregulation of antioxidant proteins, whereas mouse embryo fibroblasts with mutant BRCA1 resulted in downregulation of antioxidant proteins and an increased sensitivity to oxidative stress.^89^

BRCA1 is also important in gene silencing to prevent tumorigenesis. BRCA1 deficiency showed a disruption in heterochromatin silencing, where reduced heterochromatic foci and altered heterochromatin structure was observed in both mice and human cells, leading to loss of satellite DNA repression that could be restored by reintroducing BRCA1.^90^ In human breast tumours deficient for BRCA1, a significant decrease in the repression of satellite DNA was reported, where the increased satellite DNA expression promoted genomic instability and cancer progression.^90^

Another critical role of BRCA1 is in DNA repair, specifically base excision and double-stranded break repair. Accordingly, embryos with a BRCA1 deletion were characterized with a hypersensitivity to γ-irradiation.^96^ As previously mentioned, due to the brain’s vulnerability to oxidative stress and the toxicity of double-stranded break to post-mitotic neurons, these DNA repair pathways are particularly important to the brain. In human breast carcinoma cells, BRCA1 increases activity of select enzymes involved in the earlier stages of the base excision repair pathway.^97^ Indeed, a BRCA1 knockdown was accompanied by a reduction in base excision repair and impaired repair of oxidative stress-induced lesions.^97^^,^^98^ As previously mentioned, double-stranded break repair can occur following one of two pathways: homologous recombination or non-homologous end joining. In homologous recombination, the reliance on a repair template makes this pathway accessible only during replication.^88^ When a double-stranded break occurs, BRCA1 is recruited, among other proteins to promote end resection.^99^ In doing so, BRCA1 also assists in recruiting downstream proteins, such as RAD51 (DNA repair protein RAD51 homolog 1)^100^^,^^101^ to mediate strand invasion to identify homology before DNA synthesis can occur to repair the break.^102^ Outside of replication, the lack of requirement for a repair template in non-homologous end joining makes this pathway more readily available. In this pathway, a Ku70-Ku80 heterodimer recognizes and binds at the double-stranded break site.^103^ Although BRCA1 is more traditionally known to be involved with homologous recombination, evidence of BRCA1 in non-homologous end joining has also been reported. Binding of BRCA1 to Ku80 stabilizes the heterodimer at double-stranded breaks to promote non-homologous end joining fidelity.^104^ Indeed, human cells expressing mutant BRCA1 leads to an impairment in non-homologous end joining.^105^

Through various mechanisms, BRCA1 plays an important role in preventing aberrant cell cycle and gene expression, protecting against oxidative stress and repairing lesions in DNA. More importantly, its role in both homologous recombination and non-homologous end joining to achieve proper double-stranded break repair may potentially serve as an important component of DNA damage-induced senescence in the brain.

### Neurodevelopment, adulthood and senescence

To further emphasize the functional importance of BRCA1, mice with homozygous *BRCA1* mutations result in embryonic lethality, establishing BRCA1’s crucial role in development.^106–108^ It was found that mice homozygous for BRCA1 mutated in exon 11, the largest exon in BRCA1 spanning over half of the coding region,^107^ resulted in neurodevelopment abnormalities, specifically in the neural tube and neuroepithelium.^106^ In the brain, neuronal BRCA1 expression appears to decrease throughout development to adulthood. During development, BRCA1 is highly enriched in the neuroepithelium, whereas during adulthood, BRCA1 is enriched particularly in more actively proliferating areas, such as neural stem cell niches.^109^ Mice with an embryonic BRCA1 knockout selective for neural progenitors during development resulted in reduced overall brain volume, as well as defects in the neocortex, hippocampus and cerebellum, structures important to cognition and motor learning.^110^ Researchers determined that in the absence of BRCA1, an upregulation of p53-mediated apoptosis occurs, resulting in the proliferative defects seen in the developing brain.^110^ As development progresses, BRCA1 expression appears to decrease, with expression more limited to regions populated by proliferating neural progenitor cells. For instance, BRCA1 expression in the cerebellum is present at the external granule layers where neuronal precursor cells are situated. However, as cells migrate and differentiate to form the internal granule layer, becoming post-mitotic, only few remaining cells express BRCA1.^109^ Indeed, neural stem cells taken from the brain stem of embryonic and adult rat brain both exhibited BRCA1 expression, but differentiation of neural stem cells led to a loss of BRCA1 expression.^109^ These studies suggest that neuronal BRCA1 is associated with proliferation rather than differentiation, and that its expression in the adult brain may be decreased in mature neurons, but more highly expressed in regions populated by neural stem cells.

In addition to neural development, BRCA1 functions to maintain neuronal function in the adult brain as well. Specifically, BRCA1 is involved in maintaining neuron morphology, where BRCA1 knockdown in the dentate gyrus resulted in reduced cell size and dendritic branching compared to controls.^84^ Moreover, these knockdown cells also exhibited impaired action potential firing and long-term potentiation, suggesting that BRCA1 functions in promoting synaptic plasticity involved in learning and memory.^84^ Formation of transient double-stranded breaks in the brain has also been identified in response to neuronal activity^84^ and to regulate expression of neuronal early-response genes.^111^ Neuronal double-stranded breaks were detected to be most abundant in the dentate gyrus,^45^ with a BRCA1 knockdown in the dentate gyrus leading to impaired spatial learning and memory.^84^ Given BRCA1’s function in double-stranded break repair, its involvement could be speculated in the repair of these transient double-stranded breaks, further supporting its role in learning and memory.

Although BRCA1’s involvement in senescence specifically in the brain is less studied, loss of BRCA1 has been reported in other tissues and cell types to promote senescence. For instance, loss of BRCA1 in mammary epithelial cells induced upregulation of markers associated with senescence that led to a functional decline in mammary stem cells.^112^ In addition, mammary epithelial cells also exhibited elevated DNA damage, increased DNA damage response signalling and senescent-associated morphological changes.^113^ Alternatively, a loss of BRCA1 in human lung fibroblasts promoted increased formation of senescent-associated heterochromatic foci accompanied by an upregulation of senescent markers.^114^ Collectively, these studies demonstrate the role of BRCA1 deficiency in promoting senescence due to impaired genomic stability, however, its effects in the brain remain to be investigated.

BRCA1 functions throughout development, adulthood and ageing, where its loss can disrupt many critical cellular processes and negatively impact tissue function (Fig. 1). With ageing, where senescence becomes more prevalent and the risk for neurodegeneration increases, levels of BRCA1 also appear to decrease, likely causing reduced genomic stability. In accordance with this, BRCA1 has been implicated in the neuropathology associated with several neurodegenerative diseases, which will be highlighted in the following section.

### Pathways to neurodegeneration

Previous research has clearly identified BRCA1 and its importance in both the development and maintenance of the central nervous system. However, with age and in response to brain-related insults, BRCA1 dysregulation can negatively impact brain structure and function to promote neurodegeneration. With DNA damage as a recognized feature associated with neurodegeneration, although specific mechanisms are unclear, BRCA1 as a DNA repair protein has been implicated in several neurodegenerative diseases.

As previously mentioned, mice deficient of BRCA1 in the brain exhibited increased neuronal double-stranded breaks and identifiable cognitive deficits, both of which were exacerbated in an Alzheimer’s disease mouse model.^84^ Researchers suggested that this increase in neuronal double-stranded breaks was due to an aberrant activation of NMDA (N-methyl-d-aspartate) receptors that triggered proteasomal degradation of BRCA1 levels.^45^ In patients with Alzheimer’s disease and mild cognitive impairment, BRCA1 appeared to be depleted in hippocampal regions compared to control patients, and was instead enriched at histopathological legions.^84^ Similarly, BRCA1 has been reported to mislocalize from the nucleus and colocalize with tau at lesions associated with multiple tauopathies in neurons of disease brains, an observation that was not seen in control brains.^115^^,^^116^ Interestingly, this BRCA1 colocalization was not seen with alpha-synuclein or TARDBP (TAR DNA-binding protein) inclusions, histopathological features associated with Parkinson’s disease and amyotrophic lateral sclerosis, respectively.^116^ Rather, in a mouse model of amyotrophic lateral sclerosis, *BRCA1* expression did not appear dysregulated in motoneurons, but an upregulation of *BRCA1* was identified in microglia.^117^ Accordingly, BRCA1 expression in microglia was upregulated in human amyotrophic lateral sclerosis spinal cord samples compared to controls.^117^ In striatal cell lines modelling Huntington’s disease, BRCA1 recruitment to sites of DNA damage was impaired due to an imbalance in active and inactive BRCA1 levels, resulting in delayed DNA repair and persistent double-stranded break accumulation from a disruption in the signalling pathway.^118^ Overall, this evidence not only supports a dysregulation of BRCA1 in neurodegeneration, but suggests that its dysregulation in neurons and glial cells may vary between different neurodegenerative diseases.

Although neurons are post-mitotic, cell cycle re-entry can occur following defective DNA repair. In fact, re-initiation and dysregulation of the cell cycle is a recognized feature of neurodegenerative disorders,^119^^,^^120^ where dysregulation can occur from various stressors, such as excitotoxicity, oxidative stress and DNA damage.^121^ This process can induce senescence or apoptosis depending on the extent of cell cycle progression and the amount of DNA damage present.^122^ More specifically, progression from G0 (Gap 0) to G1 (Gap 1) phase has been associated with promoting non-homologous end joining for double-stranded break repair, however, prolonged duration in cell cycle arrest with persistent DNA damage due to improper repair may induce cellular senescence.^123^ On the other hand, continued progression into S phase (synthesis phase) has been associated with apoptosis due to added replicative stress.^122^ Studies have shown that DNA damage induction into re-entry of the cell cycle in neurons stimulates expression of cell cycle proteins and initiates DNA replication, ultimately leading to expression of apoptotic proteins.^120^^,^^121^ Accordingly, inhibiting cell cycle progression can prevent neuronal cell death by attenuating apoptotic signalling and entry into S phase,^122^ suggesting cell cycle inhibitors may be neuroprotective.^121^

In a study investigating how the clinical presentation of Alzheimer’s disease is influenced by DNA damage and cell cycle dysregulation, researchers found that more severe manifestations of Alzheimer’s disease were associated with reduced DNA repair, in addition to increased cell-cycle progression and cell death in the hippocampus.^124^ Alzheimer’s disease subjects with both cognitive impairment and Alzheimer’s disease pathology showed higher expression of markers indicative of early and late cell cycle stages, lower expression of cell cycle inhibitors, and elevated rates of apoptotic neurons compared to subjects with only disease pathology and control subjects without cognitive impairment and disease pathology.^124^ Mislocalization of BRCA1 to the cytoplasm, previously identified in several models of tau-dependent neurodegeneration, was also found to be higher in subjects with both clinical and pathological Alzheimer’s disease compared to those with only disease pathology and controls.^124^ Furthermore, BRCA1 overactivation has been associated with driving neuronal death associated with Alzheimer’s disease by promoting cell cycle re-entry.^125^ Together, this evidence further reinforces the role of BRCA1 in proper DNA repair and cell cycle regulation, and how it can impact cognition and pathology when dysregulated.

It is unclear how BRCA1 dysregulation contributes more broadly to neurodegeneration as it seems differentially dysregulated across multiple neurodegenerative diseases. Although BRCA1 expression can stimulate apoptosis by initiating cell cycle re-entry in neurons associated with Alzheimer’s disease,^125^ BRCA1 can also promote cell cycle arrest and inhibit cell cycle progression into S-phase in human cancer cells.^126^ Depending on the specific cell type or subcellular localization, BRCA1 may function in a variety of ways given its involvement in multiple cellular processes. With regards to neurodegeneration, BRCA1 dysregulation may act to induce neuronal cell death by initiating cell cycle re-entry, leading to neuronal loss and cognitive decline. Alternatively, it may act to induce DNA damage-induced senescence in neurons or glial cells without proper DNA repair, leading to structural and functional decline in the brain without a loss of neurons. Indeed, BRCA1 loss in a mice model of Alzheimer’s disease resulted in increased neuronal double-stranded breaks without an increase in neuronal apoptosis.^84^ Thus, future studies aimed at investigating BRCA1’s association with neurodegeneration would be of interest to further elucidate specific underlying mechanisms driving neurodegeneration.

## BRCA1: its role in sex-specific differences associated with neurodegeneration

Further adding to the complexity of the pathophysiology associated with neurodegeneration, sex differences have been reported in the incidence, progression and outcomes of neurodegenerative diseases.^127^^,^^128^ For instance with Alzheimer’s disease, women experience more disability and worse cognitive deterioration, while men experience worse comorbidity and higher mortality.^129^^,^^130^ Alternatively, for diseases such as Parkinson’s diseases and multiple sclerosis that involve motor function impairment, disease progression is more rapid in men, but incidence of multiple sclerosis is higher in women.^131^^,^^132^ Moreover, for Huntington’s disease, females experience worse symptoms and faster disease progression compared to males.^133^ Even outcomes resulting from other insults to the brain that increase risk for neurodegeneration, such as stroke or mild traumatic brain injury, exhibit sex differences. Following stroke, the likelihood of reoccurrence is higher in women compared to men.^127^ With mild traumatic brain injury, women often report more severe post-concussive symptoms, worse outcomes, and experience longer recovery times compared to men.^134–136^ However, despite these differences in outcomes between males and females, females are often underrepresented in neuroscience research and even when represented, sex differences are frequently overlooked,^137–139^ thereby limiting development of effective treatment strategies for brain injury and neurodegeneration.

Although specific mechanisms underlying these sex differences are currently unclear, several mechanisms have been proposed, such as differences in microglial function and oxidative stressors between males and females. Differences in immune presence that impact levels of neuroinflammation have been reported, where elevated levels of oestrogen appear to confer an enhanced immune response in females, exhibiting neuroprotective effects by reducing activated glial populations and attenuating neuroinflammation.^140^^,^^141^ Microglia in aged female rats have also been reported to be more effective in protecting against damage following cerebral ischaemia compared to those in males.^142^ However in a mouse model of Alzheimer’s disease investigating how sex and apolipoprotein E (APOE) genotype influences microglial interactions and disease pathology, females exhibited higher levels of microglia and amyloid burden compared to males expressing either APOE3 or APOE4 genotypes.^143^ On the other hand, oxidative stress is another mechanism proposed to underlie the sex differences associated with neurodegeneration. In both clinical and animal studies, males have been reported to experience more oxidative stress compared to females due to testosterone acting as an oxidative stressor.^144^^,^^145^ Moreover, endogenous testosterone has been associated with cognitive impairment particularly under increased oxidative stress.^146^ However, the difference in oxidative stress burden appears diminished when the comparison is made with menopausal women, reiterating potential neuroprotective effects elicited by oestrogen.^147^ Although males may experience more oxidative stress, exposing cells to mild levels of oxidative stress as a form of preconditioning has been shown to be neuroprotective against subsequent insults.^148^ Therefore, thresholds may exist where oxidative stress induced by testosterone may be beneficial at lower levels, but toxic when in excess.

Although it is unclear how causative these mechanisms may be, hormonal differences between males and females are consistently presented as contributors of sex-specific differences, with oestrogen harbouring neuroprotective effects.^149^^,^^150^ Challenging this view, oestrogen metabolites have been reported to induce double-stranded breaks in human breast cells through the formation of DNA adducts that result in replication fork stalling and collapse, thereby promoting genomic instability.^151^ Given the role of BRCA1 in double-stranded break repair, BRCA1-deficient cells exhibited increased levels of oestrogen-induced DNA damage, suggesting that BRCA1 is involved in repairing DNA damage induced by oestrogen metabolites.^151^ Moreover, BRCA1 is also involved in regulating oestrogen metabolism by repressing oestrogen metabolizing enzymes, where expression of these enzymes is increased in BRCA1-depleted cells leading to elevated levels of oestrogen metabolites.^151^ Therefore, BRCA1 may be involved in both preventing DNA damage by oestrogen metabolites but also its repair. Indeed, oestrogen is a well-characterized inducer of BRCA1 expression, indicating some sort of regulatory feedback mechanism.^152^ This evidence suggests that not only do females experience an additional source of DNA damage throughout their reproductive years when oestrogen levels are more elevated, but that subsequent consequences are likely exacerbated in *BRCA1* mutation carriers. In a recent study, oestrogen metabolites were shown to mediate senescence of hypothalamic astrocytes in culture, exhibiting an increase in DNA damage and senescence-associated markers.^153^ Although this study did not focus on other brain regions, investigating the effects of BRCA1 and oestrogen on stress-induced senescence in other cognition-associated brain regions would be of interest in the context of neurodegeneration.

Oestrogen signalling is also involved in learning and memory, where it has been reported to elicit opposing effects in cognition that differ based on which brain regions are affected. For instance, higher levels of oestrogen seemed beneficial towards hippocampal-associated tasks, but disadvantageous towards striatum-associated tasks.^154^ Furthermore following a stressful event, studies have reported that females and males utilize different brain circuitry for learning that in turn lead to a learning phenotype that is suppressed in females, but enhanced in males.^155^

Together, these studies demonstrate both neurotoxic and neuroprotective effects of oestrogen in the brain, with loss of BRCA1 contributing to oestrogen neurotoxicity. Although neuroprotective effects of oestrogen have been studied in depth, the continuous sex differences associated with neurodegeneration suggests other pathways may be at work to drive worse outcomes in females. With the capacity to induce cellular senescence and drive tissue dysfunction, DNA damage accumulation and the role of BRCA1 in oestrogen metabolite-induced DNA damage would be an interesting avenue to explore in its ability to initiate stress-induced senescence, and how that contributes not only to neurodegenerative pathology but also sex-specific differences.

## Conclusion

Cellular senescence is becoming increasingly recognized as a feature of neurodegeneration as a process that promotes chronic tissue dysfunction and functional decline. Stress-induced senescence, in particular, has been documented to occur following excess DNA damage and defective DNA repair, and has been observed to even precede toxic protein aggregation in several neurodegenerative diseases. Evidently, the onset of senescence within various cell types in the brain interfere with proper structure and function, contributing to impaired cognition over time. Given the functional importance of BRCA1 in the brain as a key DNA repair protein in maintaining genomic stability, several studies have affirmed its association to stress-induced senescence and neurodegeneration. Together these studies emphasize a need for future research involving BRCA1, as it will not only provide further insights into DNA damage-induced senescence, but also introduce novel therapeutic avenues against neurodegenerative diseases and associated sex-specific differences.

## Competing interests

The authors report no competing interests.

## Data availability

This review article did not involve the generation of new data.

## Abbreviations

APOE =: apolipoprotein E
ATM =: ataxia-telangiectasia mutated
BRCA1 =: breast cancer type 1
BRCT =: BRCA1 C Terminus
G0 =: Gap 0
G1 =: Gap 1
MRE11 =: meiotic recombination 11
MRN complex =: meiotic recombination 11/DNA repair protein RAD50/Nijmegen breakage syndrome 1 complex
NBS1 =: Nijmegen breakage syndrome 1
NMDA =: N-methyl-D-aspartate
p21 =: cyclin-dependent kinase inhibitor 1
p53 =: tumour protein p53
RAD50 =: DNA repair protein RAD50
RAD51 =: DNA repair protein RAD51 homolog 1
RING =: really interesting new gene
SA-*β*-gal =: senescence-associated beta-galactosidase
SIRT6 =: sirtuin 6
Sphase =: synthesis phase
TARDBP =: TAR DNA-binding protein
